# Bridging the Gap in Smoking Cessation: Unveiling Tobacco Harm Reduction in Pakistan Through Print Media Content Analysis

**DOI:** 10.1155/bmri/3822509

**Published:** 2025-03-26

**Authors:** Abdul Hameed, Daud Malik

**Affiliations:** Department of Research and Development, Alternative Research Initiative, Islamabad, Pakistan

**Keywords:** e-cigarettes, safer nicotine product, smoking cessation, tobacco control, tobacco harm reduction

## Abstract

This paper provides an in-depth content analysis of safer smoking alternatives in Pakistani print media during a 5-year period (2018–2022). The mainstream print media in the English and Urdu languages is grappling with understanding the importance of tobacco harm reduction and, as a result, the safer nicotine alternatives as one of the options to address combustible smoking. Largely, the perception portrayed by the English and Urdu newspapers through news stories, articles, and so on regarding tobacco harm reduction and safer nicotine alternatives is negative. This may be due to the strong opposition to safer nicotine alternatives by the organizations working on tobacco control in Pakistan. However, on the other hand, there has been positive coverage of safer nicotine products, with the main message around how they can help adult smokers reduce harm to their health and ultimately quit smoking. Clearly, the print media need more awareness and education regarding tobacco harm reduction and safer nicotine alternatives. The emphasis should be on providing evidence-based information about safer nicotine alternatives and their role in addressing the issue of combustible smoking prevalence.

## 1. Background

Tobacco kills up to 50% of smokers. Every year, more than 8 million people die because of tobacco use, and among them, 1.3 million are nonsmokers who are exposed to secondhand smoke. About 80% of the world's 1.3 billion tobacco users live in countries with lower or middle incomes [1]. Pakistan is one of them, with 31 million tobacco smokers. Of them, 17 million smoke cigarettes. Smoking caused 163,360 deaths in Pakistan in 2017 [2]. Almost half of the smokers (49%) in Pakistan are between 25 and 41 years old, and 38% are between 45 and 64 years old [3]. Extremely limited cessation services are available in the country. Only less than 3% of smokers successfully manage to quit in a year. Pakistan has a law to control smoking in enclosed places, but it does not say much about helping people quit smoking [4]. In 2019, the cost of diseases and deaths caused by smoking was 615.07 billion rupees ($3.85 billion). Most of this cost (70%) is because of the impact of smoking on people's productivity and the economy, not just direct medical expenses [5].

In the last two decades, a number of tobacco control measures have been introduced. These include increased taxes on cigarettes, warnings on cigarette packs, a ban on smoking in public places, and not allowing cigarette sales near schools. In 2022, Pakistan increased taxes on locally made cigarettes by 150%. Though this was mostly because of economic problems, it is still seen as a step to reduce smoking [2].

However, smoking remains a major public health issue. Pakistan has an ambivalent policy toward safer nicotine alternatives. E-cigarettes, nicotine pouches, and heat-not-burn products are legally imported and sold in a regulatory vacuum. Organizations working on tobacco control call for banning them. Tobacco harm reduction has not been given the attention it deserves in Pakistan's tobacco control debate and initiatives [6].

Harm Reduction International (HRI) defines harm reduction as measures aimed at minimizing the adverse health, social, and economic consequences of legal and illegal psychoactive drug use, without necessarily reducing drug consumption [7]. THR entails transitioning from conventional smoking to less harmful alternatives like vapes, heated tobacco products, or snus-style oral items, backed by substantial evidence of their reduced health risks [8].

In 1971, Russell emphasized nicotine as the key factor driving smoking habits. He advocated for minimizing tar levels while maintaining nicotine content in low-tar cigarettes in a 1976 paper. Russell et al. in 1980 introduced noncombustible nicotine consumption as a safer alternative to smoking [9–11]. The World Health Organization's (WHO) tobacco regulatory committee acknowledged in 2009 that certain smokeless tobacco products, particularly low-nitrosamine options like Swedish snus, pose considerably less risk than cigarettes [12]. Today, THR is regarded as the substitution of high-risk tobacco products, such as combustible cigarettes, with lower risk alternatives like nicotine replacement therapy, low-nitrosamine smokeless tobacco, and e-cigarettes [13].

Over the past five years, Pakistan has witnessed a steady increase in the use of safer nicotine alternatives, especially e-cigarettes and nicotine pouches. Today, the country has approximately 500-odd e-cigarette outlets across the country. The users, typically aged 18–35 and residing in urban areas, tend to be well-educated and financially sound. E-cigarettes are legally imported, though lacking specific policies. The government imposes taxes on their import. Despite a gradual increase in THR product users, primarily e-cigarettes, their numbers are small compared to traditional tobacco users. THR products are relatively expensive and accessible mostly in affluent localities, operating in a regulatory void. Users often lack medical guidance, making the transition from conventional smoking to THR a personal choice. Many smokers are unaware of THR product availability and learn about it from friends, driven by curiosity [14].

The print media plays a central role in agenda setting. Research has shown that news stories influence attitudes, behaviors, and policies, while letters to the editors and op-ed articles highlight issues in the form of debate and views. They may also be seen as a strategy for educational purposes, advocacy, and focused interventions [15]. However, the role of media is more significant in forming opinions in low- and middle-income countries (LMICs). This is mainly because media becomes a cost-effective tool in promoting public health behaviors and attitudes. The media coverage of safer smoking alternatives such as electronic nicotine delivery system (ENDS), alternative nicotine delivery system (ANDS), and the concept of THR is on the rise globally and locally. The coverage in the mainstream print media is critical as it shapes public perception of these alternatives [16].

## 2. Materials and Method

### 2.1. Data

A total of 10 mainstream newspapers, six in English and four in Urdu, were monitored from 1 January 2018 to 31 December 2022. These newspapers are available in almost every part of the country. As the use of safer nicotine products started in Pakistan in 2015–2016, therefore, we want to see how the mainstream print media projected their use. The selection criterion was the publishing of news, articles, letters to the editor, blogs, and so on regarding the safer nicotine products and how they were presented. All the newspapers are mainstream national dailies with countrywide coverage. Dawn, The News, The Express Tribune, The Nation, Daily Times, and Pakistan Observer are the English-language newspapers, while Jang, Nawa-e-Waqt, Dunya, and Express are the four Urdu national dailies selected for this study. Predefined thematic areas and indicators were assessed, including news related to THR, the categorization of THR news as positive or negative, and the sources of the news.

### 2.2. Method

This study employed a mixed-method approach, integrating quantitative and qualitative methods, to comprehensively explore news related to THR. The study followed an explanatory sequential design, a mixed-methods approach that commences with quantitative data collection and analysis, followed by a qualitative phase to provide insights and explanations for the quantitative findings. Two independent researchers conducted the quantitative content analysis and qualitative thematic analysis. Both researchers utilized Microsoft Excel and STATA Version 17.1 to establish predefined themes and indicators for subsequent data curation and entry.

A four-member research team was responsible for collecting data from newspaper archives, adhering to the predefined coding and thematic areas. Four journalists with more than 15–25 years of experience were employed for this study. One of them has served as editor of an Urdu daily in Islamabad. The leading journalist worked with an international organization for more than a decade exclusively for translating the news from Urdu to English. He would also analyze the translated news. These four journalists were properly trained regarding the thematic areas of the research. Additionally, one of the authors has worked with the leading newspapers of Pakistan, with a background in research on tobacco control. The translations by the journalists on the theme of the research were checked and verified. The research team was given a comprehensive overview of tobacco use in Pakistan, along with the use of safer nicotine products. They were also briefed about the regulatory framework of smoking and vaping in Pakistan. As stated above, the team comprised experienced journalists who understood how the news are published and projected. Additionally, the study included an “other specify option” for coding and thematic areas that did not fit within the predefined categories, which was managed by two senior research administrators. This rigorous data management process was crucial for ensuring the quality of the information.

For quantitative analysis, the study employed simple descriptive and cross-sectional analyses to present the data numerically. For qualitative analysis, a thematic exploration approach was used to examine information that could not be quantified but held significant importance for the overall analysis.

## 3. Results

A total of 238 news items, articles, letters to the editors, blogs, and editorials were published over the five-year period. Most of these were published in The News, one of the leading English dailies in Pakistan, followed by 13.5% and 12.2% in The Express Tribune and Dawn—other leading English dailies. Among Urdu dailies, Jang led the publication of news items regarding safer alternatives (10.5%), followed by daily Dunya (5.5%). Evidently, the English dailies have given more space to news regarding safer alternatives than the Urdu dailies (see Table 1).

Most of the news items regarding safer alternatives have been published on the city pages—nearly half of them (43.7%), followed by magazines (12.6%), letters to the editor (8%), and articles (6.3%). Less than 1% of the news regarding safer alternatives has made it to the front pages of the 10 newspapers reviewed (see Table 2).

News stories, articles, and blogs regarding safer alternatives have been given prominent space, especially in the English dailies. Most of the news stories and articles have been published in two (24%), four (19.3%), and more than four columns (23.5%). This shows that the newspapers, as and when they publish news stories about safer alternatives, give them a good display (see Table 3).

Most of the stories have been published from dateline Islamabad (39.5%), followed by Rawalpindi (6.3%) and Karachi (6.3%). The city of Karachi has more than 140 vape shops, while the twin cities of Rawalpindi and Islamabad have around 100 vape shops. The interest in the safer alternative seems to be limited to metropolitan cities (see Table 4).

Most of the news stories, articles, and blogs published about safer alternatives have been in the English newspapers. In the period under review, a little more than three-fourths (77.31%) of the published material is in English newspapers. Most of the printed material was about electronic cigarettes, commonly called e-cigarettes. The term “tobacco harm reduction” has been published and discussed in 16.4% of the news stories, articles, and blogs, followed by ENDS (12.6%), ANDS (8.8%), and HRPs (harm reduction products) (9.7%). The selected English and Urdu newspapers also published news, articles, and letters to the editor about nicotine patches and pouches, JUUL vape, NRT, and smokeless tobacco (see Table 5).

Overall, the perception about the safer smoking alternatives in the print media is negative. Only 84 or 35.2% of the stories, articles, editorials, letters to the editors, and blogs were deemed positive in their tone. These stories highlighted two aspects—safer alternatives help to reduce combustible smoking (23.8%) and also in quitting (25%). Similarly, 16.7% of the encouraging printed material in the 10 selected newspapers focused on the safer alternatives' role as smoking cessation tools. The positive stories focused on harm reduction, cessation, and quitting (see Table 6).

Almost two-thirds of the printed material in the 10 newspapers over the five-year period was deemed negative or discouraging. Most of the negative perceptions were about the health risks the safer alternatives can cause (37.4%), followed by the regulatory vacuum and avoidance of taxes (14.2%) and the addiction caused by nicotine (8.4%). There have been calls for banning safer alternatives in Pakistan, mostly by organizations working on tobacco control (see Table 7).

**Figure 1:**
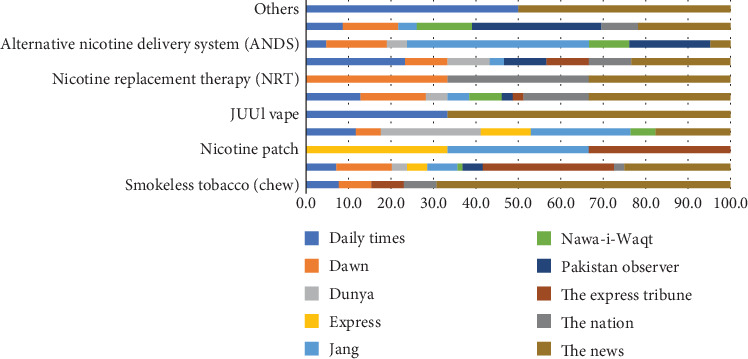
Tobacco harm reduction news by newspapers. *Source:* Author's calculation.

When the newspapers were reviewed individually, The News covered the THR issues most (26.5%) in the period under review. The focus of stories, articles, editorials, letters to the editor, and blogs was on smokeless tobacco, JUUL vape, THR, NRT, and HRPs. On the other hand, Dawn, the leading English daily in Pakistan, focused on NRT, THR, e-cigarettes, ENDS, ANDS, and HRPs. Jang, the leading Urdu daily and a sister publication of The News, published the most news stories on safer alternatives, mainly focusing on nicotine patches and pouches (see Figure 1).

**Figure 2:**
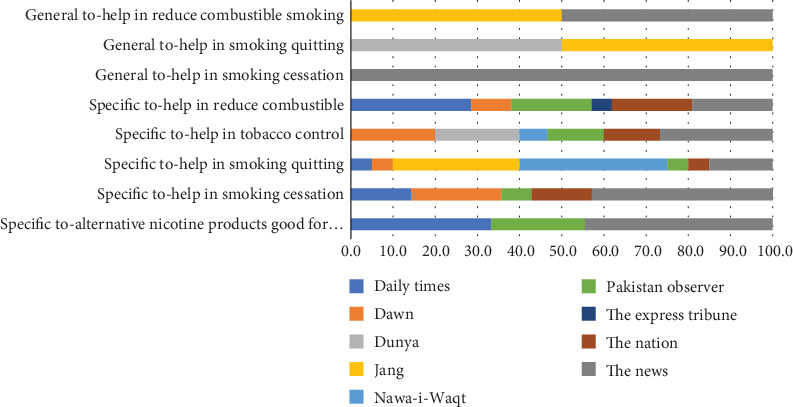
Tobacco harm reduction encouraging news by newspapers. *Source:* Author's calculation.

Most of the encouraging material regarding safer alternatives has been published in The News (27.4%), followed by 14.3% in Daily Times and 10.7% each in Dawn and The Express Tribune—all four English newspapers. The positive perception news in The News revolved around safer alternatives good for health, help in smoking cessation, and tobacco control. In Dawn, the focus was on the positive role of safer alternatives in smoking cessation and tobacco control. Jang and Nawa-e-Waqt, two leading Urdu dailies, focused on the THR's role in reducing combustible smoking and helping smokers quit the habit (see Figure 2).

**Figure 3:**
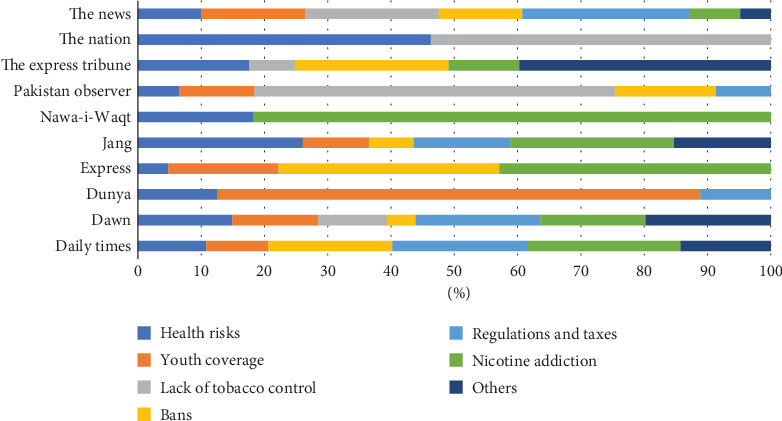
Tobacco harm reduction discouraging news by newspapers. *Source:* Author's calculation.

The News also published the most news stories, articles, editorials, and letters to the editor, which have negative connotations regarding safer alternatives. The main apprehensions revolved around youth using these products (31.3%), lack of tobacco control (40%), and regulatory vacuum (50%). The newspaper also focused on health risks because of the use of safer alternatives and nicotine addiction. The negative stories, articles, editorials, and letters to the editor in Daily Dawn were about health risks, youth getting hooked on these products, lack of clarity around regulations, and nicotine addiction. However, in Jang, most of the negative news stories, articles, and editorials were focused on health risks and the addiction caused by nicotine (see Figure 3).

These highlighted a range of efforts, concerns, and activities of organizations opposing the concepts of ANDS like e-cigarettes, vaping, smokeless tobacco, and HRPs along with the THR themes in Pakistan. These organizations fear that these products' potential risks may harm public health, particularly among the youth. They argue these products, promoted by the tobacco industry as alternatives to smoking, are not safe. They point out the increase in the use of e-cigarettes and vaping among youth, maintaining this use may lead to a new epidemic of tobacco addiction. They see this as another ploy of the tobacco industry to attract younger users.

The government's moves to regulate and tax these products are met with mixed reactions. Some organizations express concerns that imposing a tax on e-cigarettes might inadvertently legitimize their usage. Others call for stricter measures such as complete bans on the import, export, and sale of these products. They also call for an alignment with the WHO Framework Convention on Tobacco Control (FCTC) and the implementation of stricter policies to control the use of these emerging tobacco products under the FCTC regulations. They highlight the importance of the media's role in raising awareness regarding smoking cessation.

On the other hand, the organizations that support safer nicotine alternatives highlight the role of e-cigarettes, vaping, smokeless tobacco, and THR products as critical to ensuring adult smokers have more options to reduce harm to their health and ultimately quit this habit. Interestingly, these organizations back the government of Pakistan's measures for tobacco control, which include the implementation of MPOWER under FCTC.

They have repeatedly made calls in the mainstream print media that THR can help Pakistan decrease smoking prevalence and set itself on the road to being smoke-free. They stress the effectiveness of harm reduction in reducing smoking prevalence and advocate for its inclusion in Pakistan's National Tobacco Control Policy, drawing from successful models in other nations. The efforts of these organizations collectively highlight the potential benefits of harm reduction strategies, the need for policy integration, and the urgency of addressing smoking-related harm.

**Figure 4:**
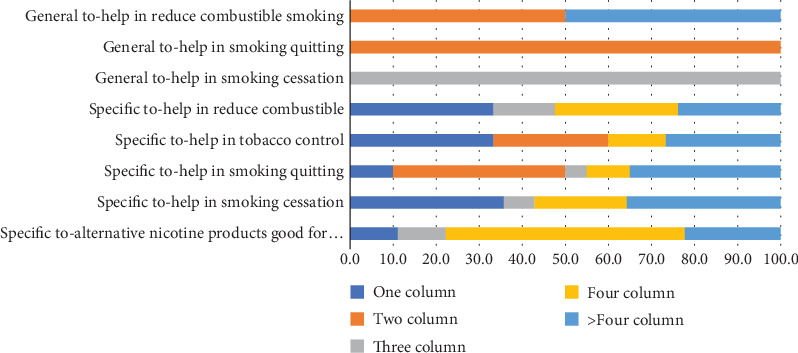
Tobacco harm reduction encouraging news by size. *Source:* Author's calculation.

Most of the positive news stories and articles have been published in more than four-column displays, indicating a good display for them. These stories and articles were regarding alternative products helpful in cessation, quitting, tobacco control, and reducing combustible smoking. The one-column positive news stories highlighted the fact that safer alternatives can help in tobacco control and subsequently reduce combustible smoking, which would ultimately contribute to smoking cessation (see Figure 4).

**Figure 5:**
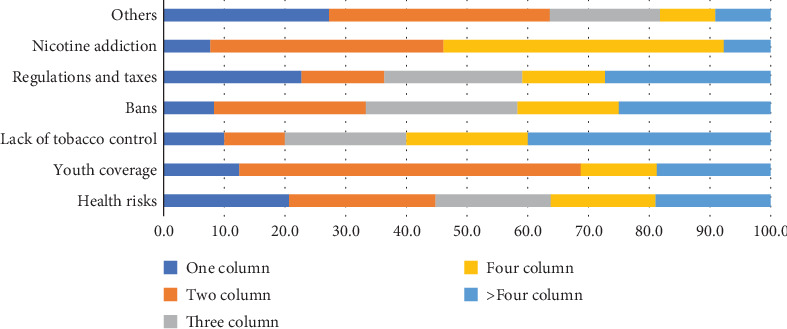
Tobacco harm reduction discouraging news by newspapers. *Source:* Author's calculation.

The news stories, articles, and letters to the editors with negative perceptions mainly revolve around risks to health. These have been published in one, two, three, four, and more than four-column displays, indicating the apprehensions about the use of alternatives to combustible smoking. The worry that youth would be hooked on these products has mainly been given a two-column display (56.3%). Similarly, the declaration that the introduction of these products would be damaging to tobacco control efforts has been given more than four-column displays (40%). The fear that these products would promote nicotine addiction has mainly been expressed in four-column stories and articles (46.2%) (see Figure 5).

## 4. Discussion and Conclusions

Evidently, English newspapers see the emergence and the use of safer nicotine alternatives as a more public health issue than the Urdu dailies. The six English dailies exhibited a significantly higher level of interest by publishing 77.31% of the news regarding safer nicotine alternatives. On the other hand, the four selected Urdu newspapers' shares were only 23%. It is important to note that the number of news stories in Urdu dailies is much more than in their English counterparts. Keeping in view the trend of publishing hundreds of news stories daily, it can be said that safer nicotine alternatives remain a nonissue for the Urdu dailies. As most of the stories, both in English and Urdu dailies, have been published on inside pages, currently, for the print media, the emergence and the use of safer nicotine alternatives remain a city-level issue.

Moreover, the qualitative data and the author's discussion with a media expert indicate that a majority of the news articles were disseminated by organizations receiving funding from the THR opposition. Specifically, the opposition has allocated substantial funding for tobacco control, exemplified by a recent $160 million program in collaboration with international organizations. This initiative aims to combat flavored e-cigarettes, and it appears that a similar narrative has been mirrored in the Pakistani media through support from these funded organizations.

Geographically, the majority of stories were published from major cities like Islamabad, Rawalpindi, and Karachi. This highlights the concentration of interest and coverage in metropolitan areas, suggesting a regional bias toward urban locales where a higher number of vape shops are present. The data underscores the prevalent negative perception of safer smoking alternatives in the Pakistani print media. A significant portion of the coverage focuses on health risks associated with these alternatives, regulatory concerns, and the fear of addiction caused by nicotine. Conversely, positive stories emphasize harm reduction, smoking cessation, and the potential benefits of using safer alternatives. There is a consensus among health experts that e-cigarettes and heated tobacco products are not fully risk-free. They highlight concerns about the harmful effects of nicotine on brain development. Additionally, they question the accuracy of claims that these products help smokers quit, urging more research to establish causal relationships [14].

The 20th century witnessed a staggering toll on human lives due to tobacco use, claiming an estimated 100 million individuals—a death toll surpassing the combined casualties of both world wars [17]. What makes this more disconcerting is that a significant portion of these smokers were unaware of the profound harm they were inflicting upon themselves. This emphasizes the critical need for harm reduction strategies within the realm of tobacco control. THR has emerged as a crucial approach to mitigating harm without completely eradicating tobacco and nicotine use [18]. The fundamental principle underlying THR is to minimize mortality and morbidity associated with tobacco consumption. It is viewed as a matter of human rights, ensuring that all smokers, regardless of their desire or ability to quit, are provided with avenues to reduce the harms caused by tobacco use [19–22].

Scientific research has consistently shown that reducing nicotine levels to about 95% of those found in conventional cigarettes could significantly benefit public health [23]. However, a considerable challenge remains as many individuals struggle to quit smoking, and global smoking cessation policies have proven inadequate in effectively addressing this issue. This challenge is reflected in Pakistan as well, where both smokers and doctors often lack the knowledge and means to effectively facilitate smoking cessation [6, 14]. Alarmingly, fewer than 3% of smokers manage to quit within a year.

Pakistan has laws in place to control smoking in enclosed spaces, yet they fall short of providing substantial assistance to individuals looking to quit [4]. Consequently, many smokers, bereft of medical guidance, are left to make the personal choice to transition from conventional smoking to THR products. Compounding this issue is the lack of awareness among smokers about the availability of THR products, often learning about them through social circles and driven by curiosity [14]. This underscores the pressing need for comprehensive public health strategies to increase awareness, provide guidance, and support individuals in making informed choices for harm reduction.

## 5. Recommendations

Based on the print media content analysis, this study suggests the following policy recommendations:

• There is a need to work with the dailies in the Urdu language regarding the use of safer nicotine alternatives as a public health issue. This can be done by contributing articles in the Urdu newspapers on the efficacy of reduced-risk products.
• For both English and Urdu newspapers in Pakistan, the use of safer nicotine alternatives remains a nonissue. Most of the news has been published on the inside pages. There is a need to approach journalists working on health issues to brief them on the importance of reduced-risk products, especially in terms of ending smoking in Pakistan.
• There is a need to provide journalists working on health issues with updated information regarding scientific developments vis-à-vis the use and regulation of safer nicotine alternatives. This may be backed by changes in laws on reduced-risk products in developed countries.

## Figures and Tables

**Table 1:** Newspaper coverage.

**Newspaper name**	**%**	**N**
Daily Times	10.9	26
Dawn	12.2	29
Dunya	5.5	13
Express	2.9	7
Jang	10.1	24
Nawa-i-Waqt	4.2	10
Pakistan Observer	8.0	19
The Express Tribune	13.5	32
The Nation	6.3	15
The News	26.5	63
Total	100	238

*Note: Source:* Authors' calculation.

**Table 2:** News coverage by page.

**News publish page**	**%**	**N**
Front page	0.8	2
Editorial page	2.5	6
Article page	6.3	15
Letters to the editor page	8.0	19
City page	43.7	104
National page	5.0	12
Magazine	12.6	30
International	14.7	35
Others page	6.3	15
Total	100	238

*Name: Source:* Authors' calculation.

**Table 3:** News coverage by column.

**News size**	**%**	**N**
One column	19.3	46
Two columns	24.0	57
Three columns	13.9	33
Four columns	19.3	46
Greater than four columns	23.5	56
Total	100	238

*Note: Source:* Author's calculation.

**Table 4:** News coverage by dateline.

**Dateline**	**%**	**N**
Islamabad	39.5	94
Lahore	0.8	2
Rawalpindi	6.3	15
Karachi	5.9	14
Multan	2.9	7
Toba Tek Singh	0.4	1
Gujranwala	0.4	1
Mardan	0.4	1
Wazirabad	0.8	2
Dera Ghazi Khan	0.4	1
International	14.7	35
Not clear	27.3	65
Total	100	238

*Note: Source:* Authors' calculation.

**Table 5:** News regarding tobacco harm reduction.

**THR coverage**	**%**	**N**
Smokeless tobacco (chew)	5.5	13
Electronic cigarettes (E-Cigs)	35.3	84
Nicotine patch	1.3	3
Nicotine pouch	7.1	17
JUUL vape	1.3	3
Tobacco harm reduction (THR)	16.4	39
Nicotine replacement therapy (NRT)	1.3	3
Electronic nicotine delivery system (ENDS)	12.6	30
Alternative nicotine delivery system (ANDS)	8.8	21
Harm reduction products (HRPs)	9.7	23
Others	0.8	2
Total	100.0	238

*Note: Source:* Authors' calculation.

**Table 6:** Positive news regarding tobacco harm reduction.

**Encouraging news**	**%**	**N**
Specific to alternative nicotine products good for health	10.7	9
Specific to help in smoking cessation	16.7	14
Specific to help in smoking quitting	23.8	20
Specific to help in tobacco control	17.9	15
Specific to help in reducing combustible smoking	25.0	21
General to help in smoking cessation	1.2	1
General to help in smoking quitting	2.4	2
General to help in reducing combustible smoking	2.4	2
Total	100.0	84

*Note: Source:* Author's calculation.

**Table 7:** News regarding tobacco harm reduction discouraging.

**Discouraging news**	**%**	**N**
Health risks	37.4	58
Youth coverage	11.0	17
Lack of tobacco control	6.5	10
Bans	15.5	24
Regulations and taxes	14.2	22
Nicotine addiction	8.4	13
Others	7.1	11
Total	100.0	155

*Note: Source:* Author's calculation.

## Data Availability

The data can be obtained from the corresponding author upon request.
